# Pharmacological Activity of Honeybush (*Cyclopia intermedia*) in Boar Spermatozoa during Semen Storage and under Oxidative Stress

**DOI:** 10.3390/ani10030463

**Published:** 2020-03-10

**Authors:** José Luis Ros-Santaella, Martin Kadlec, Eliana Pintus

**Affiliations:** Department of Veterinary Sciences, Faculty of Agrobiology, Food and Natural Resources, Czech University of Life Sciences Prague, Kamýcká 129, 165 00 Praha 6-Suchdol, Czech Republic; kadlecmartin@af.czu.cz

**Keywords:** *Cyclopia intermedia*, lipid peroxidation, oxidative stress, semen storage, sperm function

## Abstract

**Simple Summary:**

Nowadays, pig breeding is mostly conducted by artificial insemination using diluted semen stored for 1 to 5 days. During semen handling and storage, sperm quality usually declines, mainly because of oxidative stress and bacterial contamination. As cheap and natural sources of antioxidants, medicinal plants have become an alternative to the most common additives used in semen extenders. In this regard, several indigenous plants from Southern Africa have shown pharmacological activity in different animal cell types, although their effects on sperm cells have not been explored extensively. In the present study, we tested the effects of honeybush (*Cyclopia intermedia*) aqueous extract as a preservative of boar semen during 5 days of storage and under induced oxidative stress. Overall, this plant extract enhanced several sperm quality parameters and did not show any toxic effects. Supplementation with honeybush extract was able to improve the preservative properties of a long-term semen extender, thus confirming the beneficial use of plant extracts as natural additives for boar sperm.

**Abstract:**

In recent decades, an increasing number of ethnopharmacological studies have been dedicated to medicinal plants from South African fynbos. Among these plants, honeybush (*Cyclopia* spp.) has become a popular tea, mainly due to its healthy properties and caffeine-free status. The antioxidant, antimutagenic, and antimicrobial properties of this plant have been reported in several cell types, but its effects on reproductive function are still unknown. Here, we assessed the effects of honeybush (*Cyclopia intermedia*) on boar sperm parameters under induced oxidative stress (Fe^2+^/ascorbate) and during five days of semen storage at 17 °C without oxidative stress. In both experiments, four concentrations (200, 50, 12.5, and 3.125 µg/mL) of fermented honeybush were tested. Our results show that honeybush enhances sperm parameters, and no toxic effects were observed at any of the tested extract concentrations. Interestingly, honeybush (12.5 µg/mL) improved the sperm motility and kinetic parameters, preserved the plasma membrane integrity, and reduced the lipid peroxidation in the samples exposed to Fe^2+^/ascorbate (*p* < 0.05). In the stored samples, positive effects of honeybush on sperm parameters (motility, kinetics, acrosome, and mitochondria) were observed from 48 h until 120 h of semen storage (*p* < 0.05). Our results clearly show the protective effects of honeybush on sperm samples, thus promoting its use as a natural source of antioxidants for boar semen.

## 1. Introduction

In recent decades, indigenous medicinal plants from Southern Africa have been widely distributed worldwide, promoting an increase in ethnopharmacological studies [1]. Honeybush (*Cyclopia* spp.) and rooibos (*Aspalathus linearis*), which are closely related plants from the fynbos biome in South Africa, are some of the most popular teas, mainly due to their healthy properties and caffeine-free status [2]. Nowadays, special attention is paid to the use of honeybush as a phytotherapeutic agent, and about half of the related literature has been published in the last five years. Thus, for instance, the antioxidant, antimicrobial, and antimutagenic activities of honeybush have been tested in different cell types, with promising results [3,4]. However, the effects of honeybush on the reproductive function are still unknown, both in vivo and in vitro.

Pig breeding is mostly conducted by artificial insemination (AI) due to the several advantages that this practice offers to breeders (e.g., the selection of genetic lines, a higher reproductive performance, disease control, etc. [5]). Most pig AIs worldwide are carried out with diluted semen stored at 15–20 °C for 1 to 5 days [6]. Long-term extenders are able to preserve semen for more than four days, but they are considerably more expensive than short-term extenders because of their specific additive composition (e.g., nutrients, antibiotics, antioxidants, chelating agents, etc.). However, for some long-term extenders, it is advisable to perform the AI within three days after semen collection to prevent a reduction in the litter size [7]. Of the factors that negatively affect sperm quality during semen storage, bacterial contamination and increased levels of reactive oxygen species (ROS) are the most common [8,9]. Boar seminal plasma contains several antioxidant enzymes, and superoxide dismutase (SOD) and glutathione peroxidase (GPx) are the enzymes with the greatest activity [10]. The dilution of boar semen is a common practice, and it considerably decreases the levels of these enzymes and thus their action against the excess of ROS production during semen handling and storage. Excessive ROS levels lead to a state of oxidative stress, which can jeopardize the cell function by inducing lipid peroxidation and compromising the sperm fertilizing capacity [11]. Natural products, such as medicinal plants, could be employed as a cheap alternative to the common antioxidants used in boar semen extenders to reduce the negative effects of high levels of ROS on sperm cells. Recent studies have shown that the addition of plant extracts to extenders enhances sperm function during semen storage and in vitro fertility in boars [12,13].

The main goal of the present study was to evaluate the effects of fermented honeybush (*Cyclopia intermedia*) extract on samples of diluted boar semen. Because of its antioxidant properties [4], we hypothesized that the addition of honeybush extract to a semen extender preserves sperm quality under oxidative stress and prolongs sperm lifespan during semen storage. In the first experiment, in order to evaluate the potential of honeybush as an ROS scavenger, diluted semen samples were submitted to oxidative stress using Fe^2+^/ascorbate as an ROS inductor (hydroxyl radical, ^•^OH) that promotes lipid peroxidation. In the second experiment, we evaluated the effects of honeybush extract on sperm parameters during 5 days of diluted semen storage at 17 °C. For both experiments, we assessed the sperm motility and kinetic parameters, plasma membrane and acrosome integrity, and mitochondrial membrane potential (Δψ_m_). Additionally, as a marker of lipid peroxidation, the malondialdehyde (MDA) levels in the semen samples under oxidative stress were also determined.

## 2. Materials and Methods

All reagents were purchased from Sigma-Aldrich (Prague, Czech Republic), unless otherwise indicated. All the data generated from this study are available in the Supplementary Materials (File S1: Full dataset).

### 2.1. Preparation of the Honeybush Extract

Loose fermented honeybush was purchased from a specialized herbal shop (Oxalis, spol. s.r.o., Slušovice, Czech Republic). The extract was prepared by adding 50 mL of boiling ultrapure water to 1 g of honeybush and steeped for 10 min (stock solution: 20 mg/mL). Afterwards, the extract was filtered through a filter paper (Whatman n. 4), cooled to room temperature, and adjusted to pH 7 (initial pH 4.53). Then, the extract was aliquoted into cryotubes and frozen at −80 °C.

### 2.2. Total Polyphenol Content (TPC) and Total Antioxidant Capacity (TAC) of the Honeybush Extract

The TPC and TAC were determined spectrophotometrically (Libra S22, Biochrom, Harvard Bioscience Company, Cambridge, UK), as previously described [14,15]. Briefly, for TPC determination, 500 µL of Folin–Ciocalteau reagent (0.2 N) and 400 μL of Na_2_CO_3_ solution (7.5%) were added to 100 μL of ultrapure water (blank), gallic acid standard (10–200 μg/mL), or honeybush extract. The samples were incubated in the dark at room temperature for 2 h, and the absorbance was measured at 765 nm. The results were expressed in µg/mL (gallic acid equivalents). For the TAC assay, 20 μL of honeybush extract was added to 800 μL of reagent 1 (acetate buffer 0.4 M, pH 5.8) and 80 μL of reagent 2 (2,2′-azino-bis-(3-ethylbenzothiazoline-6-sulfonic acid) (ABTS)) in an acetate buffer (30 mM, pH 3.6). Five minutes after mixing, the absorbance was measured at 660 nm. A standard curve was established using known concentrations (0.25–2 mM) of 6-hydroxy-2,5,7,8-tetramethylchroman-2-carboxylic acid (Trolox). The TAC was expressed in mM (Trolox equivalents). For both analyses, four replicates were used, and each sample was assessed in duplicate.

### 2.3. Semen Collection and Processing

Our study did not involve animal handling because the sperm samples were purchased as AI doses from an animal breeding company (PROAGRO, Nymburk a.s., Czech Republic). Semen doses from 13 boars of two breeds (Duroc and Landrace) were used in this study. The semen was collected by the gloved hand method, filtered through gauze to remove gel particles, and diluted with a long-term (5 days) extender (SUS 5, Medi Nova, Italy). All semen doses had a minimum of 85% of motile sperm. In each work session, semen samples from three boars were pooled in order to reduce the effects of individual male variability. Afterwards, the sperm concentration was checked using a Bürker chamber and adjusted to ~15 × 10^6^ sperm/mL using the semen extender. In each work session, one aliquot of honeybush (stock solution: 20 mg/mL) was thawed at room temperature, and three serial dilutions were made using ultrapure water: 5 mg/mL, 1.25 mg/mL, and 0.3125 mg/mL. Then, for both experiments (samples with and without oxidative stress) and based on previous trials, four concentrations of honeybush extract were used: 200 µg/mL (HB1), 50 µg/mL (HB2), 12.5 µg/mL (HB3), and 3.125 µg/mL (HB4). The experiment was replicated five times using five different semen pools.

#### 2.3.1. Experiment 1: Sperm Samples under Oxidative Stress

Samples were split into six test tubes. One of them was used as a control (CTR). In the remaining tubes, oxidative stress was induced by 0.05 mM FeSO_4_ and 0.5 mM sodium ascorbate (Fe^2+^/ascorbate), as previously described [16]. One of the tubes was used as a control (CTR-ox), while the rest of the tubes were supplemented with the honeybush extract. The samples were stored for 4 h at 17 °C and then, before the sperm analyses, incubated at 38 °C for 20 min in a water bath. The CTR tube was also analyzed at 0 h (after 20 min of incubation at 38 °C).

#### 2.3.2. Experiment 2: Sperm Samples Stored at 17 °C for 120 h

In the same way, the semen samples were split into five tubes. One tube was used as a CTR (same control as that used in the previous experiment), and the remaining tubes were supplemented with four concentrations of honeybush, as previously described. The sperm analyses were run at 0 h (CTR only), 2 h, 48 h, and 120 h of semen storage and after incubation of the samples at 38 °C for 20 min in a water bath.

### 2.4. Sperm Motility

A sperm aliquot (5 µL) was loaded into a pre-warmed Spermtrack chamber (depth: 20 µm; PROISER, Paterna, Spain). The sperm motility and kinetic parameters were evaluated using a Computer Assisted Sperm Analysis (CASA) (NIS-Elements, Nikon, Tokyo, Japan and Laboratory Imaging, Prague, Czech Republic), which consists of an Eclipse E600 tri-ocular phase contrast microscope (Nikon, Tokyo, Japan), equipped with a warming stage set at 38 °C (Tokai Hit, Shizuoka, Japan), and a DMK 23UM021 digital camera (The Imaging Source, Bremen, Germany). The analysis was carried out using a 10× negative phase-contrast objective (Nikon, Tokyo, Japan). A total of eight descriptors of sperm motility parameters were recorded: the total motility (TM, %), average path velocity (VAP, μm/s), curvilinear velocity (VCL, μm/s), straight-line velocity (VSL, μm/s), amplitude of lateral head displacement (ALH, μm), beat-cross frequency (BCF, Hz), linearity (LIN, %), and straightness (STR, %). The standard CASA settings were as follows: frames per second, 60; minimum of frames acquired, 31; and VAP ≥ 10 µm/s to classify a spermatozoon as motile. A minimum of 200 motile sperm cells were analyzed per sample.

### 2.5. Sperm Plasma Membrane Integrity, Acrosomal Status, and Mitochondrial Activity

Plasma membrane integrity was evaluated as previously described [17], with minor modifications. Briefly, the sperm samples were incubated with propidium iodide (stock solution: 0.5 mg/mL in phosphate-buffered saline, PBS), carboxyfluorescein diacetate (stock solution: 0.46 mg/mL in dimethyl sulfoxide, DMSO), and formaldehyde solution (0.3%) for 10 min at 38 °C in the dark. Then, the spermatozoa were assessed under epi-fluorescence microscopy (Nikon Eclipse E600, Nikon, Japan; 400× magnification), and those with a complete green fluorescence over the head were considered to have an intact plasma membrane. For the acrosomal status, the percentage of sperm with a normal apical ridge (NAR [18]) was determined as previously described [16,19]. Briefly, the sperm samples were fixed in a glutaraldehyde solution (2%) and evaluated under phase contrast microscopy (400× magnification). The mitochondrial membrane potential (Δψ_m_) was evaluated as previously described [20], with minor modifications. Briefly, aliquots of the sperm samples were incubated with rhodamine 123 (5 mg/mL in DMSO) and propidium iodide (0.5 mg/mL in PBS) for 15 min at 38 °C in the dark. After that, the samples were centrifuged at 500 g for 5 min, the supernatant was removed, and the sperm pellet was resuspended in PBS. Then, the spermatozoa were evaluated using epi-fluorescence microscopy (400× magnification), and the spermatozoa showing a bright green fluorescence over the midpiece were considered to have a high Δψ_m_. Two-hundred sperm cells were assessed per analysis by the same observer.

### 2.6. Lipid Peroxidation (Sperm Samples under Oxidative Stress)

The lipid peroxidation was assessed using the thiobarbituric acid reactive substances (TBARS) assay, as previously described [16,21]. After 4 h of incubation at 17 °C, the sperm aliquots were collected and stored at −80 °C, until the analysis. The absorbance of each sample was then measured by spectrophotometry at 532 nm. A standard curve was established using known concentrations (0.5–32 µM) of 1,1,3,3-tetramethoxypropane (malondialdehyde, MDA). The levels of lipid peroxidation are shown as the μmol of MDA per 10^8^ spermatozoa. The assay was run in duplicate for each sample.

### 2.7. Statistical Analyses

The statistical analyses were carried out by the SPSS 24 statistical software package (IBM Inc, Chicago, IL, USA). Shapiro–Wilk and Levene’s tests were used to check the data normality and homogeneity of variance, respectively. To check for differences between the HB treatments in the TPC and TAC, one-way ANOVA and Tukey post-hoc tests were used. Repeated measures ANOVA (TM, NAR, and mitochondrial activity) or Friedman (plasma membrane integrity) tests were used to check for differences among sperm parameters in the CTR group during semen storage. A generalized linear model (GZLM) was used to analyze the effects of the treatments and storage times on the sperm variables. The data are expressed as the mean ± standard deviation. The statistical significance was set at *p* < 0.05.

## 3. Results

### 3.1. TPC and TAC of the Honeybush Extract

**Figure 1:**
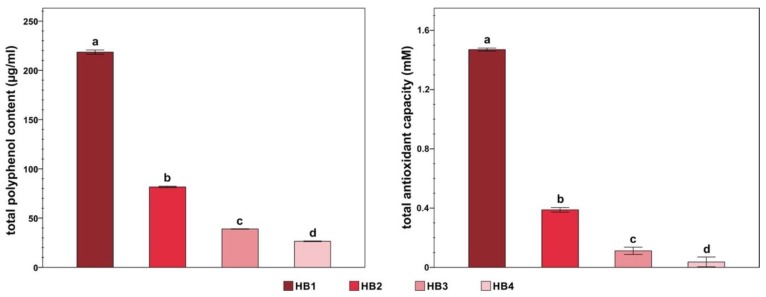
Total polyphenol content and total antioxidant capacity of the honeybush aqueous extracts. Different letters indicate significant differences between treatments (*p* < 0.01). The total polyphenol content is expressed as gallic acid equivalents. The total antioxidant capacity is expressed as Trolox equivalents. HB = honeybush. Treatments = HB1 (20 mg/mL); HB2 (5 mg/mL); HB3 (1.25 mg/mL); HB4 (0.3125 mg/mL). The data are shown as the mean ± standard deviation of four replicates.

The TPC and TAC of the honeybush extract at the different concentrations are shown in Figure 1. Overall, the TPC and TAC range from 26.56 to 218.43 µg/mL of gallic acid equivalents and from 0.04 to 1.47 mM of Trolox equivalents, respectively.

### 3.2. Effects of the Honeybush Extract on Sperm Parameters

#### 3.2.1. Experiment 1 (Samples under Oxidative Stress)

##### Sperm Motility and Kinetic Parameters

The oxidative stress induced by Fe^2+^/ascorbate remarkably reduced TM (~50% less) and several kinetic parameters in the CTR-ox samples, compared to the CTR (without oxidative stress) group (Table 1). In general, the honeybush extract cushioned the negative effects of oxidative stress on the sperm motility and kinetic parameters (Table 1). Thus, the samples treated with honeybush showed a higher TM and greater VAP, VSL, ALH, BCF, and LIN than the CTR-ox group (*p* < 0.05). For instance, the TM and VAP of the HB2 treatment were 43.4% (*p* = 0.004) and 34.8% (*p* = 0.021) higher than those of the CTR-ox group, respectively. Interestingly, all of the HB treatments did not show significant differences in VSL, when compared to the CTR group (*p* > 0.05). On the other hand, all of the samples under oxidative stress showed higher values of BCF, LIN, and STR than the CTR group (*p* < 0.001), which is probably because of their lower values of VAP, VCL, and ALH, compared to the CTR samples (*p* < 0.05).

##### Sperm Plasma Membrane and Acrosome Integrity

**Figure 2:**
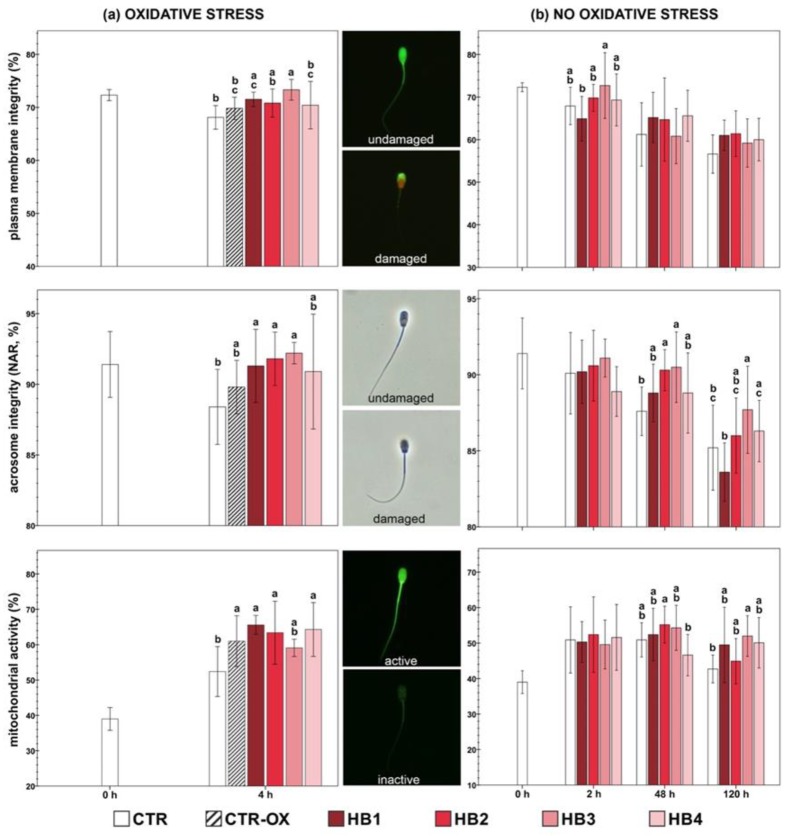
Effect of honeybush extract on the plasma membrane integrity, acrosome integrity, and mitochondrial activity of boar sperm under induced oxidative stress (**a**) and during semen storage without oxidative stress (**b**). Different letters indicate significant differences between treatments within each given time (*p* < 0.05). CTR = control samples without oxidative stress; CTR-OX = control samples under induced oxidative stress; HB = honeybush; Treatments = HB1 (200 µg/mL); HB2 (50 µg/mL); HB3 (12.5 µg/mL); HB4 (3.125 µg/mL). The data are shown as the mean ± standard deviation of five replicates.

There were no differences between the CTR group and the CTR-ox group in the sperm plasmalemma integrity (*p* > 0.05; Figure 2a). On the other hand, the HB3 treatment showed a better plasma membrane stability than the CTR-ox group (*p* = 0.013). Moreover, the HB1 and HB3 treatments showed a higher percentage of spermatozoa with an intact plasma membrane, when compared with the CTR group (*p* < 0.05). While we did not find significant differences between the CTR-ox and the HB treatments in acrosome integrity, it tended to be higher in the HB3 treatment (*p* = 0.089; Figure 2a). In addition, three HB treatments (HB1, HB2, and HB3) showed a higher percentage of intact acrosome than the CTR group (*p* < 0.05).

##### Mitochondrial Activity

There were no significant differences between the HB treatments and the CTR-ox group in the Δψ_m_ (*p* > 0.05). Unexpectedly, all of the samples under oxidative stress (except HB3) showed a higher percentage of spermatozoa with high Δψ_m_ than the CTR group (*p* < 0.05; Figure 2a).

##### Lipid Peroxidation

**Figure 3:**
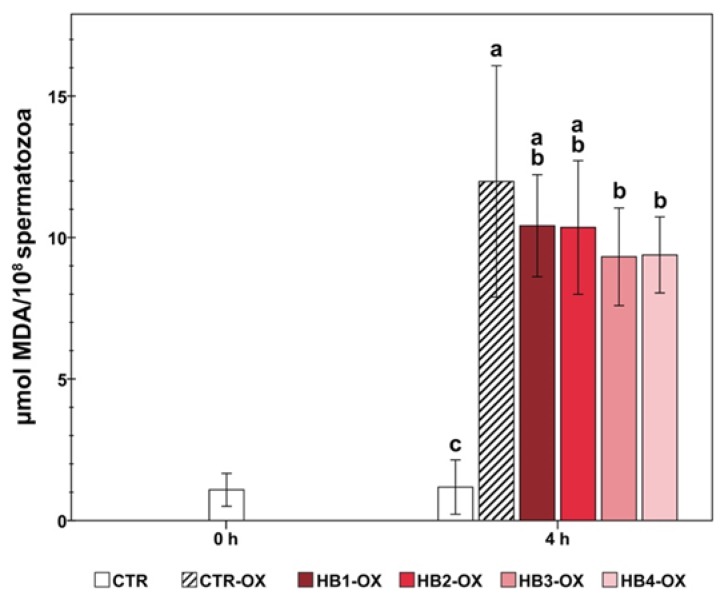
Effect of honeybush extract on the lipid peroxidation in boar sperm samples under oxidative stress (except CTR). Different letters indicate significant differences between treatments (*p* < 0.05). MDA = malondialdehyde; CTR = control samples; OX = samples under induced oxidative stress; HB = honeybush; Treatments = HB1 (200 µg/mL); HB2 (50 µg/mL); HB3 (12.5 µg/mL); HB4 (3.125 µg/mL). The data are shown as the mean ± standard deviation of five replicates.

The addition of Fe^2+^/ascorbate provoked a significant increase in the MDA levels in all samples, compared to the CTR group (*p* < 0.001; Figure 3). On the other hand, the HB3 and HB4 treatments showed lower levels of lipid peroxidation than the CTR-ox group (*p* = 0.027 and *p* = 0.031, respectively).

#### 3.2.2. Experiment 2 (Samples Stored for 120 h, without Oxidative Stress)

##### Sperm Motility and Kinetic Parameters

The effects of honeybush on the motility and kinetic parameters of boar spermatozoa are shown in Table 2. During semen storage, the CTR group showed a decrease in TM from 0 h to 120 h (*p* < 0.05). By contrast, the TM of the HB3 treatment did not differ, at any incubation time, from the CTR group at 0 h (*p* > 0.05). Positive effects of honeybush on the sperm motility and kinetic parameters were observed from 48 h until 120 h of semen storage. Thus, the HB treatments showed a higher TM at 48 h (HB2) and at 120 h (HB2, HB3, and HB4) of semen storage, when compared to the CTR group (*p* < 0.05). Furthermore, at 120 h, the HB treatments showed greater values of VAP (HB3) and VCL (HB3 and HB4) than the CTR group (*p* < 0.05). On the other hand, at 2 h of semen storage, there was a significant decrease in LIN and STR in the HB3 treatment, when compared with the CTR group (*p* < 0.01), but these treatments did not differ at the subsequent storage times. At 120 h, the HB4 treatment showed a lower STR than the CTR group (*p* = 0.038), which is probably due to the higher value of VAP compared to the CTR group (*p* > 0.05).

##### Sperm Plasma Membrane and Acrosome Integrity

In the CTR samples, the percentage of spermatozoa with an intact plasma membrane dropped from 72.30 ± 1.04% at 0 h to 56.60 ± 4.51% at 120 h (*p* < 0.01). While several HB treatments had higher values of sperm plasma membrane integrity at different incubation times, they were not significantly different from those of the CTR group (*p* > 0.05; Figure 2b). During semen storage, the acrosome integrity of the CTR group decreased from 91.40 ± 2.33% at 0 h to 85.20 ± 2.80% at 120 h (*p* < 0.01). The honeybush treatments showed a higher percentage of spermatozoa with an intact acrosome at 48 h (HB2 and HB3) and at 120 h (HB3) in comparison with the CTR group (*p* < 0.05; Figure 2b).

##### Mitochondrial Activity

During semen storage, there were no significant differences in the Δψ_m_ of the CTR group between 0 h and 120 h (*p* > 0.05). At 120 h, the HB3 treatment showed a higher Δψ_m_ in comparison with the CTR samples (*p* = 0.021, Figure 2b). The remaining treatments also showed a higher Δψ_m_ when compared with the CTR group, but the differences were not significant (*p* > 0.05).

## 4. Discussion

In the present study, we investigated the effects of honeybush extract on boar sperm parameters under oxidative stress and during semen storage for up to 120 h. This is the first study that uses honeybush extract as a source of natural antioxidants for sperm cells. Overall, the supplementation with honeybush was able to improve the preservative properties of a long-term extender for boar semen. Our results clearly show a positive effect of this plant extract on a wide set of sperm parameters, which are linked to fertility indicators in sows (non-return rate, pregnancy rate, farrowing rate, and litter size) [22]. The positive effects on the sperm parameters during semen storage (i.e., sperm motility and kinetic parameters, acrosome integrity, and mitochondrial activity) were observed from 48 h until 120 h. On the other hand, the honeybush also preserved the sperm motility, kinetic parameters, and plasma membrane integrity and reduced the lipid peroxidation in the sperm samples under oxidative stress. Based on our findings, the most suitable concentrations of honeybush extract for boar semen range from 3 to 50 µg/mL (TPC: 0.3–0.8 µg/mL gallic acid equivalents), both during long-term storage and under oxidative stress. The present study confirms the beneficial use of plant extracts as natural preservatives for boar semen.

We found that the suitable concentrations of honeybush for boar semen are similar to those of rooibos [12], although the latter shows a higher TPC and TAC, which is supported by other studies [2,23]. These differences in the TPC between the plant extracts suggest that the protective effects of honeybush on boar sperm may be related to its polyphenol profile, rather than the TPC per se. While they are closely related plants, the polyphenol composition of honeybush is different from that of rooibos [23]. Thus, the positive effects on sperm performance could be linked to some of the main polyphenols found in honeybush tea (e.g., hesperidin and luteolin), which have protective effects on male reproductive function [24,25]. On the other hand, the content of chemical elements in these South African plants shows huge differences, with some of them (e.g., K and P) being more abundant in honeybush than in rooibos [26].

The ROS generating system employed in this study promotes a decrease in sperm motility and kinetic parameters, together with an increase in MDA levels in the semen samples, as previously reported [16]. Our results clearly show that the honeybush extract mitigates the negative effects of oxidative stress on boar spermatozoa. Thus, the HB3 treatment (12.5 µg/mL) simultaneously preserves the sperm motility, kinetic parameters, and plasma membrane integrity and reduces the MDA content in the semen samples exposed to Fe^2+^/ascorbate. In agreement with our results, Petrova et al. [27] reported a protective effect of honeybush against UVB-induced skin damage, with a reduction of MDA levels, in mice. On the other hand, the Δψ_m_ of all the samples submitted to oxidative stress was higher, compared with the CTR group without oxidative stress. Similarly, it has been reported that this ROS generator does not affect the Δψ_m_ during semen incubation in a wild ungulate (red deer) [28]. In our study, the Fe^2+^/ascorbate promotes an acute reduction in the sperm motility and kinetic parameters (mainly VAP and VCL), which can lead to a lower ATP consumption, and this may increase the Δψ_m_ (energy storage). Nevertheless, the involvement of Δψ_m_ in several cellular processes remains rather obscure and requires further investigation [29].

Most pig AIs are conducted with extended semen and performed within 48 h after the semen collection [6,30]. In this regard, honeybush extract preserves sperm motility and acrosome integrity after 48 h of semen storage and also shows a protective effect on sperm mitochondria and kinetic parameters (VAP and VCL) at 120 h. Even though a long-term extender (up to 5 days) was used, we detected a significant decrease in sperm motility from 0 h to 120 h in the CTR group. Interestingly, the honeybush extract (HB3) maintained this sperm parameter unaltered until 120 h of semen storage, showing no differences compared with the CTR group at 0 h. Only the HB1 treatment (200 µg/mL) showed neither positive nor detrimental effects, when compared with the CTR samples, at the different incubation times, although some sperm parameters were enhanced but not significantly. In a previous study [12] using a related plant (rooibos) and at a similar concentration (170 µg/mL), we found a significant decrease in the percentage of motile sperm in comparison with the CTR group at 96 h of semen storage. These differences could be due to the higher TPC found in the rooibos extract. In this regard, some amounts of ROS are necessary for normal sperm function [11], so that a high polyphenol concentration in long-term semen storage could be detrimental due to excessive ROS scavenging activity. On the other hand, the decrease in sperm performance observed at 120 h of semen storage (CTR group) could be related to the proliferation of some contaminant bacterial genera, which appear to thrive in extended boar semen [31], given their resistance to the most commonly used antibiotics [32,33]. It has recently been reported that honeybush aqueous extract exerts antimicrobial activities [3] against some of the microorganisms (e.g., *Staphylococcus* spp., *Streptococcus* spp., and *Candida* spp.) commonly found in boar semen, which decrease sperm quality, thus affecting, for instance, the litter size [34,35]. Moreover, of the polyphenols found in honeybush that may have a positive effect on sperm performance, the isoflavone formononetin stimulates in vitro fertilization rates when it is added to the sperm preincubation medium, but the mechanism of action has not yet been elucidated [36]. Further studies are necessary to elucidate the mechanisms of action of honeybush on sperm parameters, such as the possible reduction of bacterial contamination during semen storage.

## 5. Conclusions

Our results clearly show that honeybush extract exerts a protective action against the deleterious effects of oxidative stress in boar sperm cells by preserving the motility and plasma membrane integrity and reducing the MDA levels. Furthermore, during semen storage at 17 °C, the honeybush extract enhances the sperm quality (motility, acrosome integrity, and Δψ_m_) from 48 h until 120 h. The most suitable concentration of honeybush extract to be used in diluted boar semen (with or without oxidative stress) is 12.5 µg/mL. Honeybush extract represents an alternative and cheap source of antioxidants for the preservation of boar semen.

## Figures and Tables

**Table 1:** Motility and kinetic parameters of boar sperm samples submitted to oxidative stress and supplemented with honeybush extract.

Time	Treatment	TM (%)	VAP (µm/s)	VCL (µm/s)	VSL (µm/s)	ALH (µm)	BCF (Hz)	LIN (%)	STR (%)
0 h	CTR	88.95 ± 4.08	56.68 ± 7.82	105.76 ± 12.04	34.68 ± 3.92	3.73 ± 0.46	14.02 ± 0.44	34.65 ± 2.63	63.25 ± 3.83
4 h	CTR	89.82 ± 2.84 ^a^	54.72 ± 10.94 ^a^	97.93 ± 17.83 ^a^	38.02 ± 5.49 ^ab^	3.74 ± 0.60 ^a^	14.19 ± 0.45 ^a^	40.38 ± 2.92 ^a^	70.01 ± 4.76 ^a^
	CTR-ox	46.09 ± 13.03 ^b^	31.38 ± 6.80 ^b^	42.47 ± 8.23 ^b^	30.01 ± 6.41 ^c^	1.94 ± 0.35 ^b^	17.16 ± 0.52 ^b^	69.96 ± 2.98 ^b^	95.03 ± 0.81 ^b^
	HB1-ox	48.73 ± 17.38 ^b^	33.99 ± 5.40 ^bc^	44.40 ± 4.29 ^b^	32.56 ± 5.25 ^ac^	2.06 ± 0.27 ^bc^	17.78 ± 1.12 ^bc^	72.17 ± 5.54 ^bc^	95.01 ± 1.09 ^b^
	HB2-ox	**66.09 ± 12.54** ^c^	**42.30 ± 7.23** ^c^	53.98 ± 8.31 ^b^	**40.45 ± 7.01** ^bd^	**2.48 ± 0.40** ^c^	**18.40 ± 0.55** ^c^	**74.41 ± 2.47** ^c^	94.75 ± 0.97 ^b^
	HB3-ox	**61.99 ± 14.04** ^c^	39.89 ± 9.77 ^bc^	51.59 ± 12.31 ^b^	**37.97 ± 8.91** ^ad^	2.38 ± 0.53 ^bc^	**18.06 ± 0.45** ^c^	**73.66 ± 0.99** ^c^	94.72 ± 1.02 ^b^
	HB4-ox	**61.69 ± 13.70** ^c^	39.56 ± 9.44 ^bc^	52.58 ± 12.77 ^b^	**37.73 ± 8.78** ^ad^	2.43 ± 0.48 ^bc^	17.75 ± 0.35 ^bc^	71.94 ± 2.32 ^bc^	94.83 ± 0.83 ^b^

Different superscript letters indicate significant differences between treatments within each given time (*p* < 0.05). Bold numbers indicate significant differences between the control-ox group and HB treatments (*p* < 0.05). CTR = control samples; ox = samples under induced oxidative stress; HB = honeybush; Treatments = HB1 (200 µg/mL); HB2 (50 µg/mL); HB3 (12.5 µg/mL); HB4 (3.125 µg/mL). TM = total motility; VAP = average path velocity; VCL = curvilinear velocity; VSL = straight-line velocity; ALH = amplitude of lateral head displacement; BFC = beat-cross frequency; LIN = linearity; STR = straightness. The data are shown as the mean ± standard deviation of five replicates.

**Table 2:** Motility and kinetic parameters of boar sperm during semen storage at 17 °C and supplemented with honeybush extract.

Time	Treatment	TM (%)	VAP (µm/s)	VCL (µm/s)	VSL (µm/s)	ALH (µm)	BCF (Hz)	LIN (%)	STR (%)
0 h	CTR	88.95 ± 4.08	56.68 ± 7.82	105.76 ± 12.04	34.68 ± 3.92	3.73 ± 0.46	14.02 ± 0.44	34.65 ± 2.63	63.25 ± 3.83
2 h	CTR	88.25 ± 1.93	55.55 ± 7.98	98.33 ± 9.61	40.54 ± 7.21	3.63 ± 0.47	14.38 ± 0.64	41.63 ± 4.61 ^a^	72.77 ± 4.32 ^a^
	HB1	88.42 ± 4.57	57.31 ± 10.15	101.84 ± 12.97	39.56 ± 5.49	3.90 ± 0.75	14.14 ± 0.28	39.40 ± 2.27 ^ab^	69.05 ± 4.96 ^a^
	HB2	89.00 ± 3.24	58.98 ± 11.02	104.70 ± 16.35	40.64 ± 5.54	3.86 ± 0.57	14.46 ± 0.41	39.71 ± 2.31 ^ab^	69.23 ± 4.98 ^a^
	HB3	89.03 ± 3.78	58.59 ± 8.88	105.19 ± 12.44	36.89 ± 4.46	4.10 ± 1.06	14.03 ± 0.66	**35.60 ± 2.41** ^b^	**63.25 ± 4.76** ^b^
	HB4	88.96 ± 5.43	58.78 ± 10.98	104.71 ± 16.92	39.64 ± 6.03	4.01 ± 0.39	14.19 ± 0.63	38.84 ± 3.06 ^ab^	67.58 ± 4.62 ^ab^
48 h	CTR	84.55 ± 5.07 ^b^	65.47 ± 8.75	118.69 ± 13.68	48.40 ± 7.50	4.33 ± 0.47	14.20 ± 0.65	42.21 ± 3.18	73.90 ± 2.89
	HB1	88.50 ± 3.88 ^ab^	68.47 ± 7.74	126.71 ± 13.51	49.07 ± 6.32	4.58 ± 0.55	14.07 ± 0.64	40.04 ± 5.00	71.83 ± 5.09
	HB2	**90.09 ± 2.78** ^a^	68.14 ± 8.05	125.84 ± 15.38	49.37 ± 5.95	4.58 ± 0.55	13.90 ± 0.64	40.26 ± 4.55	72.05 ± 5.14
	HB3	86.71 ± 4.89 ^ab^	68.49 ± 10.30	129.23 ± 20.12	47.94 ± 8.12	4.64 ± 0.71	13.72 ± 0.64	38.21 ± 3.69	69.54 ± 3.38
	HB4	88.58 ± 5.97 ^ab^	67.97 ± 9.54	127.06 ± 17.17	46.81 ± 5.92	4.65 ± 0.66	13.67 ± 0.37	37.67 ± 3.21	68.43 ± 4.59
120 h	CTR	75.43 ± 5.18 ^b^	56.63 ± 7.98 ^b^	104.58 ± 13.60 ^b^	46.80 ± 8.66	4.32 ± 0.56	13.61 ± 0.59	45.17 ± 5.40	80.55 ± 5.95 ^a^
	HB1	79.65 ± 6.82 ^ab^	62.50 ± 5.87 ^ab^	117.03 ± 15.87 ^ab^	50.80 ± 3.49	4.45 ± 0.52	13.61 ± 0.76	44.20 ± 5.89	80.18 ± 6.11 ^ab^
	HB2	**81.14 ± 8.67** ^a^	63.81 ± 8.01 ^ab^	119.73 ± 13.71 ^ab^	50.86 ± 8.62	4.44 ± 0.49	13.55 ± 0.59	42.65 ± 5.75	77.72 ± 6.18 ^ab^
	HB3	**84.00 ± 3.59** ^a^	**66.90 ± 5.49** ^a^	**125.58 ± 12.67** ^a^	52.37 ± 6.66	4.68 ± 0.44	13.40 ± 0.65	41.96 ± 4.27	76.41 ± 5.36 ^ab^
	HB4	**82.62 ± 4.25** ^a^	66.10 ± 10.00 ^ab^	**125.54 ± 23.66** ^a^	50.56 ± 7.81	4.90 ± 0.44	13.24 ± 0.87	41.13 ± 5.37	**74.72 ± 6.12** ^b^

Different superscript letters indicate significant differences between treatments within each given time (*p* < 0.05). Bold numbers indicate significant differences between the CTR group and HB treatments (*p* < 0.05). CTR = control samples; HB = honeybush; Treatments = HB1 (200 µg/mL); HB2 (50 µg/mL); HB3 (12.5 µg/mL); HB4 (3.125 µg/mL); TM = total motility; VAP = average path velocity; VCL = curvilinear velocity; VSL = straight-line velocity; ALH = amplitude of lateral head displacement; BFC = beat-cross frequency; LIN = linearity; STR = straightness. The data are shown as the mean ± standard deviation of five replicates.

## Supplementary Materials

The following are available online at https://www.mdpi.com/2076-2615/10/3/463/s1. File S1: Full dataset.
